# Klinische Variablen und Management des disseminierten Granuloma anulare – eine monozentrische retrospektive Auswertung von 33 Fällen aus den Jahren 2021 bis 2023

**DOI:** 10.1007/s00105-024-05436-2

**Published:** 2024-12-18

**Authors:** Michael Spindler, Mark Berneburg, Konstantin Drexler, Bernadett Kurz, Julian Kögel, Dennis Niebel

**Affiliations:** Klinik und Poliklinik für Dermatologie und Allergologie, Universitätsklinikum Regensburg, Franz-Josef-Strauß-Allee 11, 93053 Regensburg, Deutschland

**Keywords:** Granuloma anulare, Granulomatöse Erkrankungen, Biologika, Small molecules, Phototherapie, Granuloma annulare, Granulomatous disease, Biologicals, Small molecules, Phototherapy

## Abstract

**Hintergrund und Ziel der Arbeit:**

Granuloma anulare (GA) ist eine nichtinfektiöse, granulomatöse Erkrankung der Haut, die meist lokalisiert und selbstlimitierend ist. 15 % der Fälle zeigen eine Disseminierung mit häufig protrahiertem Krankheitsverlauf. Ziel dieser Studie ist, das Patientenkollektiv mit disseminiertem GA an einer deutschen Universitätsklinik und die Behandlungsmodalitäten zu charakterisieren.

**Material und Methoden:**

Es erfolgte eine retrospektive monozentrische Auswertung am Universitätsklinikum Regensburg im Zeitraum zwischen 2021 und 2023 mit deskriptiv-statistischer Auswertung des Patientenkollektivs und der eingesetzten Therapieverfahren.

**Ergebnisse:**

Im Zeitraum wurden 239 Patienten mit GA identifiziert, davon zeigten 33 Patienten ein histologisch gesichertes disseminiertes GA. 25 Patienten (76 %) waren weiblich, das Durchschnittsalter lag bei 57,4 ± 14,4 Jahren. 17 Patienten (53 %) verneinten Beschwerden, häufige Symptome umfassten Dysästhesien, Juckreiz und Schmerzen. Häufige Begleiterkrankungen waren Diabetes mellitus, Schilddrüsenerkrankungen, atopische Dermatitis und koronare Herzerkrankung. Therapeutisch wurden in absteigender Häufigkeit topische Glukokortikoide, systemische Glukokortikoide, Phototherapie, topische Calcineurininhibitoren und Dimethylfumarat eingesetzt. Nur 6 Patienten (18 %) zeigten eine partielle oder vollständige Remission.

**Diskussion:**

Aufgrund fehlender zugelassener Therapien werden unzureichend wirksame Therapien bei disseminiertem GA eingesetzt. Prospektive, randomisierte, placebokontrollierte Studien sind erforderlich, um die Wirksamkeit neuartiger zielgerichteter therapeutischer Verfahren zu untersuchen.

## Abstract (en)

**Background and objectives:**

Granuloma annulare (GA) is a noninfectious, granulomatous dermatosis that is generally localized and self-limiting. In 15% of cases, the disease disseminates with protracted trajectories. This study aims to characterise the patient population with disseminated GA at a German university hospital and to explore treatment modalities.

**Materials and methods:**

A retrospective monocentric evaluation was conducted at the University Hospital Regensburg between 2021 and 2023 with descriptive statistical analysis of the patient population and the treatment modalities used.

**Results:**

During the specified period, 239 patients with GA were identified, 33 of whom had histologically confirmed disseminated GA. Of the patients, 25 (76%) were women. Average age was 57.4 ± 14.4 years. Furthermore 17 patients (53%) denied symptoms. Common symptoms included dysesthesia, itching and pain. Frequent concomitant diseases were diabetes mellitus, thyroid disease, atopic dermatitis and coronary heart disease. Topical glucocorticoids, systemic glucocorticoids, phototherapy, topical calcineurin inhibitors and dimethyl fumarate were used therapeutically in descending frequency. Only 6 patients (18%) showed partial or complete remission.

**Conclusion:**

Due to the lack of approved therapies, disseminated GA is treated with inadequately effective regimens. Prospective randomized placebo-controlled trials are needed to investigate the efficacy of novel targeted therapies.

Granuloma anulare zählt zu den relativ häufig anzutreffenden dermatologischen Erkrankungen. Ein disseminiertes Auftreten entsprechender Hautveränderungen ist zwar seltener, geht jedoch aufgrund der starken kosmetischen Beeinträchtigung mit einer erheblichen psychosozialen Belastung einher. Das Fehlen zugelassener wirksamer Therapien führt in der Praxis häufig zur Anwendung wenig wirksamer bzw. unzureichend wirksamer Behandlungen. Der vorliegende Beitrag postuliert eine Versorgungslücke bei diesem Krankheitsbild und diskutiert vor dem Hintergrund vielversprechender neuer Therapieansätze die Notwendigkeit größer angelegter placebokontrollierter Studien.

## Hintergrund und Ziel der Arbeit

**Abb. 1:**
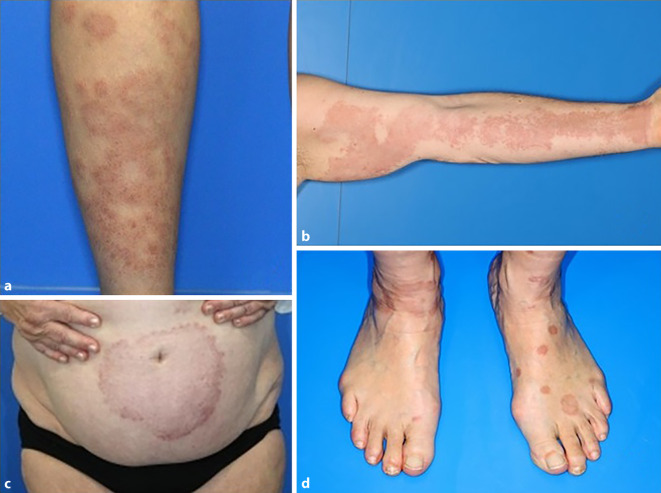
Ausgewählte klinische Bilder von Patienten mit disseminiertem Granuloma anulare. **a** Multiple, girlandenartig konfluierende, bis zu 3 cm durchmessende, erythematöse, ringförmige Plaques am Unterschenkel. **b** Flächig konfluierende, gruppiert stehende erythematöse Papeln von 5 mm Durchmesser am Unterarm, flächige, homogene, erythematöse Plaque am Oberarm. **c** 15 cm durchmessende erythematöse Plaque mit Randbetonung periumbilikal. **d** Multiple, solitär stehende, kreisrunde, bräunliche, bis zu 2 cm durchmessende Plaques am Fußrücken

Granuloma anulare (GA) ist eine entzündliche, nichtinfektiöse, granulomatöse Erkrankung der Haut [17]. Klinisch zeigt sich die Ausbildung von erythematösen oder bräunlichen Papeln und Plaques, die eine typische ringförmige Struktur mit Randbetonung ausbilden. Am häufigsten ist ein lokalisiertes Auftreten von einzelnen oder wenigen Läsionen [2]. Etwa 15 % der Patienten zeigen ein disseminiertes Auftreten mit mehr als 10 Läsionen. Diese Form der Erkrankung präsentiert sich als besonders therapierefraktär mit protrahiertem Krankheitsverlauf und stellt eine besondere klinische Herausforderung dar ([8, 24, 30]; Abb. 1).

**Abb. 2:**
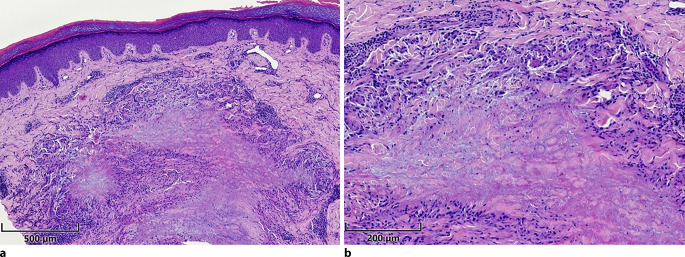
Histologischer Befund bei disseminiertem Granuloma anulare. **a** Übersicht, unauffällige Epidermis, dermal zeigt sich ein ausgedehntes Palisadengranulom mit zentraler Nekrobiosezone. **b** Detail, es dominieren Histiozyten, nur spärliche Beimischung von Lymphozyten. Jeweils Hämatoxylin-Eosin Färbung, Maßstabsbalken entspricht 500 µm bzw. 200 µm

Histologisch zeigt sich ein identisches Bild wie bei lokalisierten Formen (Abb. 2 und 3). Es kommen umschriebene nodulär aggregierte Histiozyten mit spärlichem lymphozytärem Begleitinfiltrat zur Darstellung, die ein Palisadengranulom um eine zentrale Nekrobiosezone ausbilden. Mittig im Granulom zeigt sich eine Vermehrung von Glykosaminoglykanen, die mittels Alcianblau-Färbung dargestellt werden kann. Selten stellen sich multinukleäre Riesenzellen und Beimischungen von Eosinophilen oder Plasmazellen dar, ein Erregernachweis gelingt nicht. Die Ätiologie der Erkrankung ist noch nicht ausreichend erforscht. Eine Aktivierung von Th1-, Th2- sowie Januskinase-Signalwegen wurde nachgewiesen. Assoziationen bestehen zu Diabetes mellitus, Fettstoffwechselstörungen sowie Autoimmunerkrankungen, insbesondere der Schilddrüse [22, 23].

**Abb. 3:**
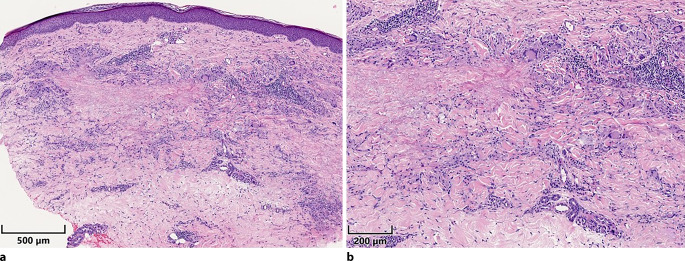
Histologischer Befund bei disseminiertem Granuloma anulare. **a** Übersicht, unauffällige Epidermis, dermal eher interstitielles granulomatöses Infiltrat. **b** Detail, hier stärkere Beimischung von Lymphozyten und mehrherdig Nachweis multinukleärer Riesenzellen. Jeweils Hämatoxylin-Eosin-Färbung, Maßstabsbalken entspricht 500 µm bzw. 200 µm

Die Datenlage zum disseminierten GA ist spärlich, Ziel dieser Studie ist es, das Patientenkollektiv mit disseminiertem GA an einer deutschen Universitätsklinik und die Behandlungsmodalitäten näher zu charakterisieren.

## Material und Methoden

Diese retrospektive und monozentrische Studie wurde an der dermatologischen Klinik des Universitätsklinikums Regensburg durchgeführt. Es wurde ein Ethikantrag bei der Ethikkommission der Universität Regensburg gestellt und bewilligt (Antrag – 23-3541-104). Das hausinterne Krankenhausinformationssystem (KIS) wurde für den Zeitraum 2021 bis einschließlich 2023 bezüglich der Diagnose „Granuloma anulare“ (ICD-10-Code L92.0) systematisch durchsucht. Es konnten insgesamt 239 Patienten identifiziert werden. Ergänzend wurden Daten aus der abteilungsinternen Fotoabteilung sowie der dermatohistopathologischen Dokumentation gewonnen. Eingeschlossen in die weitere Auswertung wurden nur Patienten mit histologisch gesichertem disseminiertem GA, definiert als Auftreten von mehr als 10 Läsionen zu mindestens einem Zeitpunkt. Letztlich konnten 33 Patienten eingeschlossen und ausgewertet werden. Die erfassten klinischen Parameter waren Alter, Geschlecht, Lokalisation der Läsionen, Größe der Läsionen, Anteil der betroffenen Körperoberfläche, Symptomatik, Maß an psychischer Belastung durch die Erkrankung, Vortherapie, aktuelle Therapie, Therapieansprechen und Begleiterkrankungen. Hinsichtlich des Therapieansprechens wurden 4 Kategorien definiert: „stabile Erkrankung“: Änderung der betroffenen Körperoberfläche um maximal 10 %; „partielle Remission“: Reduktion der betroffenen Körperoberfläche um mehr als 10 %; „vollständige Remission“: vollständige Abheilung aller Läsionen; „progressive Erkrankung“: Anstieg der betroffenen Körperoberfläche um mehr als 10 %. Statistische Analysen wurden mit IBM SPSS Statistics Version 28 durchgeführt. Tabellen und Diagramme wurden mit Graph Pad Prism Version 10 erstellt.

## Ergebnisse

### Klinische Charakteristika

Von den 33 inkludierten Patienten waren 25 weiblich (76 %) (Tab. 1). Das durchschnittliche Alter lag bei 57,4 ± 14,4 Jahren. Es zeigte sich eine betroffene Körperoberfläche von 2–35 % (Mittelwert: 9,3 % ± 8,0 %), hierbei lagen ein minimaler Läsionsdurchmesser von 0,3 cm und ein maximaler Läsionsdurchmesser von 15 cm (Mittelwert: 5,5 ± 4,6 cm) vor. 17 Patienten (53 %) verneinten Beschwerden, Dysästhesie wurde von 4 Patienten (13 %), Juckreiz von 9 Patienten (28 %), Schmerzen von 3 Patienten (9 %), Grippesymptomatik von 1 Patienten (3 %) und Schwellung von 1 Patienten (3 %) angegeben. An Begleiterkrankungen wurden dokumentiert: Diabetes mellitus (5), Hypothyreose (4), Hashimoto-Thyreoiditis (3), atopische Dermatitis (3), koronare Herzerkrankung (3), Hypercholesterinämie (2), Asthma bronchiale (2), Sarkoidose (1), Acne inversa (1), rheumatoide Arthritis (1), Hyperurikämie (1) und Mammakarzinom (1). Therapeutisch wurde bei 22 Patienten (67 %) bereits vor Vorstellung in unserem Zentrum ein topisches Glukokortikoid verwendet (keine konsistente Angabe zur Wirkstoffklasse oder zum verwendeten Präparat), 3 Patienten (9 %) erhielten vorab systemische Glukokortikoide, 2 Patienten (6 %) eine UVA-Therapie, 2 Patienten (6 %) Vitamin-E-Kapseln, 1 Patient (3 %) Dimethylfumarat, und 9 Patienten (27 %) waren zum Vorstellungszeitpunkt therapienaiv. Lediglich bei 4 Patienten (12 %) zeigte die Vortherapie eine kurzfristige leichte Verbesserung des Erscheinungsbildes.

**Tab. 1:** Klinische Charakteristika der Patientenkohorte

Geschlecht, *N* (%)	Weiblich	25	(76)
	Männlich	8	(24)
Alter, Jahre, Durchschnitt ± SD/Median (min–max)		57,4 ± 14,4	58 (26–83)
Größe der Läsion, cm, Durchschnitt ± SD/Median (min–max)		5,5 ± 4,6	4,0 (0,3–15)
Körperoberfläche, %, Durchschnitt ± SD/Median (min–max)		9,3 ± 8,0	7 (2–35)
Begleitsymptomatik, *N* (%)	Keine Beschwerden	17	53,1
	Dysästhesie	4	12,5
	Juckreiz	9	28,1
	Schmerzen	3	9,4
	Grippesymptomatik	1	3,1
	Schwellung	1	3,1
Psychosoziale Belastung	Keine Angabe	18	54,5
	Keine Beschwerden	4	12,1
	Belastung durch Erscheinungsbild	10	30,3
	Angst vor Progredienz	3	9,1
	Vermeidungsverhalten	5	15,2
Begleiterkrankungen, *N* (%)	Hashimoto-Thyreoiditis	3	9,1
	Atopische Dermatitis	3	9,1
	DM Typ II	5	15,2
	Hypercholesterinämie	2	6,1
	Koronare Herzerkrankung	3	9,1
	Hypothyreose	4	12,1
	Asthma bronchiale	2	6,1
	Sarkoidose	1	3,1
	Acne inversa	1	3,1
	Rheumatoide Arthritis	1	3,1
	Hyperurikämie	1	3,1
	Mammakarzinom	1	3,1
Vortherapie, *N* (%)	Keine Vortherapie	9	27,3
	Top. Glukokortikoide	22	66,7
	Syst. Glukokortikoide	3	9,1
	UVA-Therapie	2	6,1
	Vitamin-E-Kapseln	2	6,1
	Dimethylfumarat	1	3,1

Es gaben 10 Patienten (30 %) an, durch das optische Erscheinungsbild einen psychischen Leidensdruck zu erfahren, 3 Patienten (9 %) berichteten von einer ausgeprägten Sorge bzw. Angst vor einer möglichen (weiteren) Progredienz der Erkrankung, und 5 Patienten (15 %) berichteten von einem sozialen Vermeidungsverhalten begründet durch Scham („Baden ist für mich tabu“). Aus der Dokumentation geht hervor, dass diese Aussagen nahezu ausschließlich aktiv von den Patienten geäußert, nicht standardisiert durch den Arzt erfragt wurden. Bei 18 Patienten (55 %) wurden psychische Belastungen nicht geäußert oder nicht dokumentiert. Systematische Scores (z. B. dermatologischer Lebensqualitätsindex/DLQI) wurden nicht regelhaft eingesetzt und aufgrund der retrospektiven Datenlage liegen hierzu keine konsistenten Daten vor.

### Therapieansprechen

**Abb. 4:**
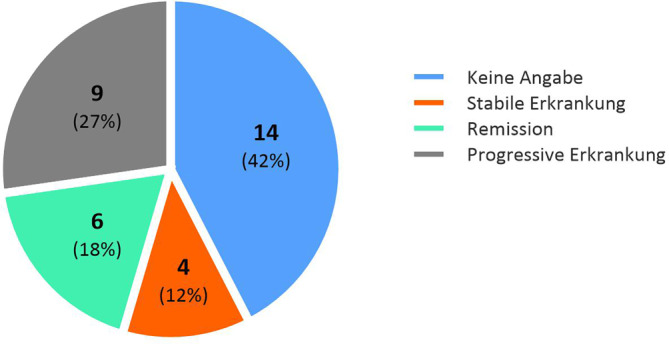
Ansprechen der Patientenkohorte auf die in unserem Zentrum eingesetzten Therapieverfahren, definiert als stabile Erkrankung (< 10 % Änderung der betroffenen Körperoberfläche), partielle oder vollständige Remission (> 10 % Verringerung der betroffenen Körperoberfläche) und progressive Erkrankung (> 10 % Zunahme der betroffenen Körperoberfläche)

**Abb. 5:**
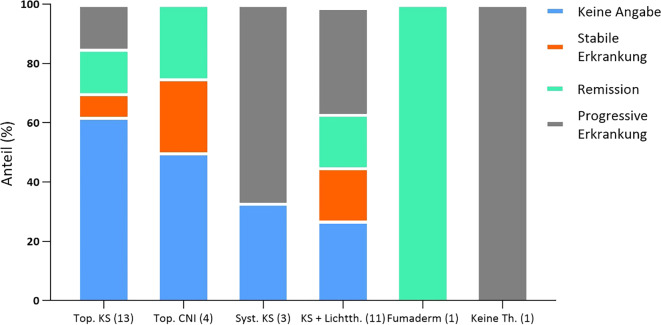
Ansprechen der einzelnen Therapieverfahren im Detail, Definitionen wie zuvor bezeichnet: *top.* *KS* topische Kortikosteroidtherapie, *top. CNI* topische Calcineurininhibitoren, *syst. KS* systemische Kortikosteroidtherapie, *KS* *+* *Lichtth.* Kortikosteroidtherapie und ergänzende Lichttherapie, *Keine Th.* keine Therapie

Insgesamt zeigten durch die erhaltene Therapie 4 Patienten (12 %) eine stabile Erkrankungsaktivität, 6 Patienten (18 %) eine partielle Remission, kein Patient eine vollständige Remission und 9 Patienten (27 %) eine progrediente Erkrankung (in 14 Fällen liegt keine Verlaufsdokumentation vor) (Abb. 4). Therapeutisch erhielten 13 Patienten in unserer Klinik ausschließlich topische Glukokortikoide (hierbei – soweit Angaben vorhanden – Präparate der Klasse III oder IV nach Niedner). Hierbei zeigte 1 Patient eine Stabilisierung der Krankheitsaktivität, 2 Patienten zeigten eine partielle Remission und 2 Patienten eine progressive Erkrankung unter der Therapie (Abb. 5). Bei 8 Patienten liegt keine Verlaufsdokumentation vor. 3 Patienten erhielten systemische Glukokortikoide, davon zeigten 2 eine progressive Erkrankung (fehlende Angaben beim 3. Fall). 11 Patienten erhielten zusätzlich zur topischen Glukokortikosteroidtherapie eine Lichttherapie (Anwendung von Bade-PUVA bei 6 Fällen, Anwendung von UVB-311-nm-Therapie bei 5 Fällen, keine konsistenten Angaben zur Anzahl der durchgeführten Sitzungen). In 2 Fällen ging dies mit einer Stabilisierung der Krankheitsaktivität einher (UVB 311 nm), in 2 Fällen mit partieller Remission (Bade-PUVA) und in 4 Fällen mit Progress der Erkrankung (2 Fälle mit UVB 311 nm, 2 Fälle mit Bade-PUVA), bei 3 Fällen fehlten weitere Angaben. Ausschließlich topische Calcineurininhibitoren (Tacrolimus) wurden bei 4 Patienten eingesetzt (Stabilisierung der Krankheitsaktivität: 1 Patient, partielle Remission: 1 Patient, fehlende Angaben: 2 Patienten). Dimethylfumarat wurde bei einem Patienten eingesetzt, bei welchem sich eine partielle Remission der Erkrankung zeigte. In einem Fall wurde keine Therapie eingesetzt, hier zeigte sich eine Progredienz der Erkrankung.

## Diskussion

### Vergleich unserer Daten mit der Literatur

Beim Geschlechtsverhältnis zeigte sich in unserer Kohorte keine wesentliche Abweichung von der Literatur [20, 24]. Das für Granuloma anulare im Allgemeinen vorbeschriebene Verhältnis von 3 zu 1 (Frauen zu Männer) traf auch auf unsere Kohorte mit disseminiertem GA zu. Das Durchschnittsalter von 57,4 Jahren entsprach ebenfalls der publizierten Datenlage [17, 24]. In der Literatur wird die Erkrankung GA meistens als asymptomatisch beschrieben, in unserer Kohorte gaben dagegen 15 von 33 Patienten Beschwerden an (45,5 %). In einer weiteren größeren Fallserie von disseminiertem GA mit 61 Patienten gaben ebenso zumindest 37 % Beschwerden an [24]. Somit muss die Sichtweise einer beschwerdearmen, somit teilweise auch bagatellisierten Erkrankung mindestens für die disseminierte Form des GA infrage gestellt werden. Das Vorliegen von Komorbiditäten wie Diabetes mellitus Typ II (15,2 %) und Hypothyreose (12,1 %) passt zu vormals publizierten Daten einer großen Kohortenstudie (5137 Individuen mit GA), dort wurde von 21 % der Patienten mit Diabetes und 14 % der Patienten mit Hypothyreose [4] berichtet. Die Aktivierung von T‑Zellen und Makrophagen sowie erhöhte Spiegel an Entzündungsmediatoren (v. a. Interleukin 6) bei Diabetes mellitus werden in der Literatur als Wegbereiter für GA postuliert [3, 25].

### Therapieansätze

Die Daten der hier vorliegenden Kohorte zeichnen das Bild einer resistenten Erkrankung. Von den 19 Patienten mit vorliegenden Angaben über das Therapieansprechen zeigte keiner eine vollständige Remission, 6 (32 %) zeigten eine teilweise Remission, 4 (21 %) eine Stabilisierung der Erkrankung und 9 (47 %) eine progressive Erkrankung. Dies ist im Einklang mit der Literatur [24, 26], wenngleich vereinzelt vielversprechende Ergebnisse in Fallserien unter Einsatz von Hydroxychloroquin (35 % Ansprechrate bei einer Serie von 26 Patienten mit generalisiertem GA), Methotrexat (60 % Ansprechrate bei einer Serie von 15 Patienten mit generalisiertem GA), Dapson und verschiedenen monoklonalen Antikörpern (z. B. Adalimumab, Dupilumab, Infliximab) beschrieben wurden [7, 14, 15, 17]. Dimethylfumarate zeigten in einigen Einzelfallberichten und 2 Fallserien teilweise vollständige Remission der Erkrankung [1, 10, 12, 19], möglicherweise könnte ein Publikationsbias vorliegen. Phototherapeutisch werden häufig UVB-Schmalspektrum (311 nm) und PUVA angewendet [6, 27]. Der Einsatz von JAK-Inhibitoren wurde wiederholt beschrieben [5, 13, 16, 28, 29]. Im ausgewerteten Kollektiv wurde bei 4 Patienten nach erfolgloser topischer Therapie mit Kortikosteroiden eine topische Therapie mit Calcineurininhibitoren durchgeführt. Da sich in einem der 4 Fälle eine Verbesserung und in einem Fall eine Stabilisierung des Hautbefundes zeigte, erscheint ein Therapieversuch mit topischen CNI nach topischen Kortikosteroiden prinzipiell als sinnvoll. In der Kohorte lagen gehäuft Erkrankungen aus dem atopischen Formenkreis vor; dies stützt Überlegungen einer überlappenden Pathophysiologie mit Überwiegen von Typ-II-assoziierten Signalwegen. Dies kann bei mittelschwerer bis schwerer atopischer Dermatitis den Einsatz von Januskinase(JAK)-Inhibitoren oder Biologika begründen, die auch für GA wirksam sein könnten. Upadacitinib im Speziellen konnte in einigen Fallberichten bei disseminiertem GA Erfolge erzielen, die Verlaufsbilder zeigten teilweise nach einigen Wochen nahezu eine vollständige Rückbildung der Läsionen [13, 28–30]. Abrocitinib und Baricitinib konnten ebenfalls in Einzelfällen eine deutliche und rasche Verbesserung der Veränderungen bei disseminiertem GA herbeiführen [18, 21]. Auch topisch konnte ein JAK-Inhibitor (Tofacitinib) bei einem Patienten mit mehreren Läsionen eines GA das Hautbild merklich positiv beeinflussen [9].

### Psychosoziale Belastung

Bisher in der Literatur wenig betrachtet, soll die vorliegende Studie auf ein erhebliches Maß der psychosozialen Beeinträchtigung durch die Erkrankung aufmerksam machen. So gaben 18 der 33 Patienten (55 %) eine entsprechende Belastung an. Es zeigte sich in der retrospektiven Auswertung der Fälle, dass vereinzelt Anträge zur Kostenübernahme für Systemtherapien bei den Krankenkassen gestellt, jedoch immer abgelehnt wurden. In einem Fall übernahm der Patient aufgrund des hohen Leidensdruckes die Kosten für eine Therapie mit Dimethylfumarat selbst, worunter es zu einer deutlichen Besserung der Erkrankung kam. Eine Veröffentlichung von 2015 erörterte die Behandlungsbedürftigkeit des disseminierten GA. Man kam zu dem Schluss, dass die Erkrankung durch Nekrobiose der Dermis eine Beeinträchtigung in objektiver Dimension (z. B. durch Störung von mechanischen Eigenschaften der Haut) darstellt. Die subjektive Dimension der Beeinträchtigung sei jedoch abgeleitet von einer erhöhten psychiatrischen Komorbidität ebenfalls vorhanden [11]. Es sollte daraus folgernd eine routinemäßige und standardisierte Erhebung der psychosozialen Belastung erfolgen (z. B. mittels DLQI-Score), welche auch eine Kostenübernahme bei Krankenkassen für vielversprechende Therapieansätze erleichtern könnte.

### Limitationen

Die vorliegenden Daten wurden monozentrisch und retrospektiv erhoben. Folglich ist die Aussagekraft aufgrund fehlender Angaben teilweise limitiert. Psychische Belastung wurde beispielsweise zwar aktiv geäußert, jedoch nicht systematisch mittels validierter Scores erfasst. Bei einigen Patienten/-innen fehlt eine Verlaufsdokumentation zur Beurteilung des Therapieansprechens. Einzelne Parameter (z. B. bei durchgeführter Lichttherapie oder angewendeten Vortherapien) wurden nicht ausreichend genau dokumentiert. Diese Aspekte sollen in prospektiven Erhebungen adressiert werden.

## Fazit für die Praxis

- Disseminiertes Granuloma anulare zeigt eine hohe Behandlungsresistenz, die psychosoziale Belastung und die Symptome werden unterschätzt.
- Mangels Zulassung und Kostenübernahme aktuell bekannter systemischer Therapieansätze besteht eine Versorgungslücke in der Behandlung des disseminierten Granuloma anulare.
- Doppelblinde, randomisierte, placebokontrollierte Studien sind erforderlich, um die Wirksamkeit neuartiger zielgerichteter therapeutischer Verfahren zu etablieren.

## Data Availability

Die Daten, die zur Unterstützung der Ergebnisse dieser Studie verwendet wurden, sind größtenteils im Manuskript enthalten und können in den Tabellen und Abbildungen gefunden werden. Zusätzliche Daten sind auf Anfrage beim Korrespondenzautor erhältlich. Manche Daten sind aufgrund von (z. B. Datenschutzbestimmungen, vertraulichen Informationen) nicht öffentlich zugänglich.
